# Bispecific T-Cell Engagers, Cell Therapies, and Other Non-Checkpoint Immunotherapies for Metastatic Uveal Melanoma: A Narrative Review

**DOI:** 10.3390/jcm15020641

**Published:** 2026-01-13

**Authors:** Jakub Kleinrok, Weronika Pająk, Joanna Pec, Kamil Rusztyn, Joanna Dolar-Szczasny, Alicja Forma, Grzegorz Teresiński, Jacek Baj

**Affiliations:** 1 Department of Correct, Clinical and Imaging Anatomy, Medical University of Lublin, ul. Jaczewskiego 4, 20-090 Lublin, Poland; klejs.90@gmail.com; 2 Department of Forensic Medicine, Medical University of Lublin, Jaczewskiego 8b, 20-090 Lublin, Poland; wapajak@gmail.com (W.P.); apec1410@gmail.com (J.P.); formaalicja@gmail.com (A.F.);; 3 Faculty of Chemistry, University of Warsaw, Ludwika Pasteura 1, 02-093 Warszawa, Poland; kamil.rusztyn12@gmail.com; 4 Department of General and Pediatric Ophthalmology, Medical University of Lublin, 20-093 Lublin, Poland

**Keywords:** metastatic uveal melanoma, bispecific T-cell engager, tebentafusp, adoptive cell therapy, tumor-infiltrating lymphocytes, oncolytic virus, HLA-A*02:01, immunotherapy

## Abstract

Metastatic uveal melanoma (MUM) remains largely refractory to immune-checkpoint inhibition, so recent research has turned to bispecific T-cell engagers (BTCEs), adoptive-cell therapies (ACTs), and oncolytic viruses (OVs). To summarize the available clinical evidence, we performed a structured literature search across PubMed, Scopus, and Europe PMC for primary studies published between 1 January 2010 and 31 May 2025 that enrolled at least three adults with MUM, treated with one of these modalities, and that reported efficacy or grade-3+ safety outcomes; two reviewers independently performed screening, data extraction, and risk-of-bias assessment, and because of notable heterogeneity, we synthesized the findings narratively. Twenty-two studies met the criteria—thirteen phase I–III trials, eight observational cohorts, and one case series—covering fifteen BTCE cohorts, four ACT cohorts, and three OV cohorts. Tebentafusp, the dominant BTCE evaluated in roughly 1150 HLA-A*02:01-positive patients, extended median overall survival from 16.0 to 21.7 months (hazard ratio 0.51, with three-year follow-up HR 0.68) in its pivotal phase-III trial despite objective response rates of only 5–12%, with early skin rash and week-12 circulating-tumor-DNA clearance emerging as consistent markers of benefit. Tumor-infiltrating lymphocyte therapy, administered to about thirty patients, produced objective responses in 11–35% and occasional durable complete remissions, although median progression-free survival remained 2–6 months and severe cytopenias were universal. Three early-phase OV studies, totaling twenty-nine patients, yielded no radiographic responses but showed tumor-specific T-cell expansion and transient disease stabilization. Safety profiles reflected the mechanism of action: tebentafusp most often caused rash, pyrexia, and usually manageable cytokine-release syndrome with grade-3+ events in 40–70% yet discontinuation in roughly 2%; TIL therapy toxicity was driven by lymphodepleting chemotherapy and high-dose interleukin-2 with one treatment-related death; and OVs were generally well tolerated with no more than 20% grade-3 events.

## 1. Introduction

Uveal melanoma (UM) is the most common intraocular cancer in adults. However, it remains rare (0.4 to 10 cases per million worldwide) with stable incidence. It occurs more frequently in males than in females. The most common primary location of UM is the choroid [1].

Localized, early-stage disease can be managed with locoregional therapies directed at the eye, such as enucleation, brachytherapy with radioisotopes (ruthenium-106 or iodine-125), and proton beam therapy. Collectively, these approaches achieve excellent local tumor control, with reported rates reaching over 90%. However, despite advances in treatment techniques, the risk of metastasis remains high [2,3]. UM carries a 15% cumulative risk of metastasis at 5 years, rising to 36% at 30 years. The youngest age cohort shows a significantly lower metastatic incidence and thus longer metastasis-free survival than the oldest cohort. Across all age categories, the liver is by far the most common first metastatic site, followed by the lung and bone [4]. The overall survival of patients with metastatic UM has long remained unacceptably short [5]. However, the introduction of new drugs in recent years has led to a greater treatment efficacy [6].

One potential treatment strategy for metastatic uveal melanoma is the use of immune checkpoint inhibitors (ICI). Three main strategies can be defined, each employing monoclonal antibodies directed against anti-programmed cell death protein 1 (PD-1), anti-programmed death ligand 1 (PD-L1), and anti-cytotoxic T-lymphocyte-associated protein 4 (CTLA-4). The outcomes of this therapy, especially when single-agent treatment was used, remain unsatisfactory. ICI combination therapy (PD-1 + CTLA-4) significantly improved outcomes, yet they remain suboptimal [7].

It is intriguing that a therapy strongly effective against cutaneous melanoma remains only modestly efficacious in uveal melanoma. This phenomenon can be associated with tumor mutational burden (TMB), which represents the total number of somatic mutations per million bases found in a specific tumor. TMB is correlated with improved responsiveness to immune checkpoint inhibitors (ICI) in many cancer types [8]. Cutaneous melanoma exhibits the highest median TMB (13.09 mutations per megabase) amongst solid tumors. By contrast, uveal melanoma displays the lowest one (0.34 mutations per megabase) [9], resulting in a low number of tumor neoantigens derived from somatic mutations. This leads to limited substrate to generate antitumor immunity [10]. Early studies indicate that pairing immunotherapy with other approaches, such as cell behavior-modifying drug therapy or radiation therapy, can offer a potential route to overcome the limited efficacy of ICI [11].

The poor clinical outcomes associated with ICI underscore the need for alternative immunotherapeutic strategies, such as BTCEs, ACTs, and OVs.

The first BTCE to receive regulatory approval was tebentafusp, a fusion molecule that combines a high-affinity T-cell receptor recognizing the gp100 peptide with an anti-CD3 domain. It redirects polyclonal T cells to lyse gp100-positive melanoma cells. Currently, it is approved only as a systemic therapy for adult HLA-A*02:01-positive patients, with unresectable or metastatic uveal melanoma [12]. Unfortunately, only about 50% of Caucasian patients express the HLA-A*02:01 allele. Consequently, tebentafusp can be offered only to a subset of patients [13]. Together with acquired resistance manifesting as disease progression after tebentafusp [14], this restriction highlights the urgent need for alternative systemic treatment strategies, especially for HLA-A*02:01-negative patients and for those who relapse following tebentafusp.

ACT is emerging as another major immunologic pillar in MUM. It includes several approaches, including tumor-infiltrating lymphocytes (TIL), T-cell receptor-engineered T cells (TCR-T), and chimeric-antigen-receptor T cells (CAR-T). Adoptive TIL transfer is a personalized cancer therapy that harnesses patient-derived, tumor-resident T cells by activating and expanding them ex vivo before reinfusion, to unleash their anti-tumor activity [15]. TCR-T therapy isolates a patient’s T cells and inserts an affinity-enhanced T-cell receptor that recognizes a tumor peptide presented by HLA-A*02:01-positive melanoma cells [16]. In contrast, CAR-T strategies bypass HLA altogether by targeting surface antigens. Several potential targets for CAR-T therapy have been identified in uveal melanoma. B7-H3 CAR-T cells are engineered to recognize the B7-H3 protein, which is expressed on nearly all UM cells. Upon binding, they trigger perforin-/granzyme-mediated destruction of UM cells, without requiring the tumor’s HLA for recognition [17].

OVs are naturally occurring or genetically engineered viruses that can selectively eliminate UM tumor cells through two complementary mechanisms: direct virus-mediated lysis and subsequent induction of antitumor immune responses [6].

## 2. Materials and Methods

### 2.1. Search Strategy

This narrative review is based on a comprehensive literature search performed across PubMed/MEDLINE, Scopus, and Europe PMC covering the period from 1 January 2010 to 31 May 2025. The search strategy combined terms related to uveal melanoma with keywords for non-checkpoint immunotherapies, including BTCEs, ACTs, and OV therapies.

The exact search string used for PubMed and Scopus was as follows:

(“uveal melanoma” OR “ocular melanoma” OR “choroidal melanoma” OR “iris melanoma”) AND (tebentafusp OR “bispecific T-cell engager” OR bispecific OR ImmTAC OR PRAME OR “tumor-infiltrating lymphocyte” OR TIL OR lifileucel OR “adoptive cell therapy” OR “T-cell receptor therapy” OR “TCR therapy” OR “CAR T” OR “CAR-T” OR “chimeric antigen receptor” OR oncolytic OR “oncolytic virus” OR T-cell receptor-engineered).

Equivalent search strategies adapted for syntax were used in Scopus.

The Europe PMC query (executed 31 May 2025) was as follows:

(((((“uveal melanoma” OR “ocular melanoma” OR “choroidal melanoma” OR “iris melanoma”) AND (tebentafusp OR “bispecific T-cell engager” OR bispecific OR ImmTAC OR PRAME OR “tumor-infiltrating lymphocyte” OR TIL OR lifileucel OR “adoptive cell therapy” OR “T-cell receptor therapy” OR “TCR therapy” OR “CAR T” OR “CAR-T” OR “chimeric antigen receptor” OR oncolytic OR “oncolytic virus”)) NOT (PUB_TYPE:(Review))) AND (HAS_FT:Y OR (HAS_FREE_FULLTEXT:Y))) AND (PUB_TYPE:(“clinical conference”) OR PUB_TYPE:(“clinical study”) OR PUB_TYPE:(“clinical trial”) OR PUB_TYPE:(“clinical trial protocol”) OR PUB_TYPE:(“clinical trial, phase i”) OR PUB_TYPE:(“clinical trial, phase ii”) OR PUB_TYPE:(“clinical trial, phase iii”) OR PUB_TYPE:(“clinical trial, phase iv”))) AND (FIRST_PDATE:[2010 TO 2025]).

Equivalent search strategies adapted for syntax were used in Scopus.

The complete search protocols and results for each database are provided in Table 1.

### 2.2. Eligibility Criteria

Studies were included if they met the following criteria:

- Population: Adults (≥18 years) with histologically confirmed MUM.
- Interventions: Any clinical trial or study involving BTCEs (e.g., tebentafusp), ACTs (e.g., TIL, CAR-T, and TCR-T), and OV therapies.
- Outcomes: Reports of clinical efficacy (e.g., objective response rate (ORR), progression-free survival (PFS), overall survival (OS)) or safety (grade ≥3 adverse events (AEs)).
- Study design: Interventional trials (phases I–III), retrospective cohort studies (e.g., analysis of patients treated with one therapy in one center), or case series (≥3 patients).
- Language: English only.
- Exclusion criteria were the following: preclinical/in vitro/animal-only studies, reviews, editorials, commentaries, protocols, and trials using ICIs as monotherapy.

All inclusion and exclusion keywords were summarized in Table 2.

### 2.3. Study Selection

All retrieved citations were imported into Rayyan.ai, a web-based platform for screening citations. Duplicate entries were automatically detected and manually verified. Two reviewers independently screened titles and abstracts. Full texts were retrieved for potentially relevant articles, and inclusion decisions were made by consensus. Discrepancies were resolved by discussion with a third reviewer.

### 2.4. Data Extraction

For all included studies, data were extracted (see Table 3) using a standardized template. Extracted variables included the following:

- Author(s), year of publication;
- Study design and clinical trial phase;
- Number of enrolled patients;
- Type of immunotherapy intervention (e.g., tebentafusp, TIL, CAR-T, TCR therapy, or OVs).

### 2.5. Data Synthesis

Due to the anticipated heterogeneity in study designs, populations, interventions, and reported outcomes, quantitative synthesis (meta-analysis) was not planned a priori. Instead, data were synthesized narratively, categorizing the findings by intervention type (BTCE, ACT, and OV). Results were summarized using structured tables, highlighting clinical efficacy (ORR, PFS, and OS) and safety (frequency of ≥grade 3 AEs).

If sufficient homogeneous data were identified for specific subgroups or interventions (e.g., tebentafusp), exploratory quantitative pooling of key outcomes, such as OS and hazard ratios (HRs), was considered using random-effects meta-analysis methods.

## 3. Results

### 3.1. Study Selection

**Figure 1:**
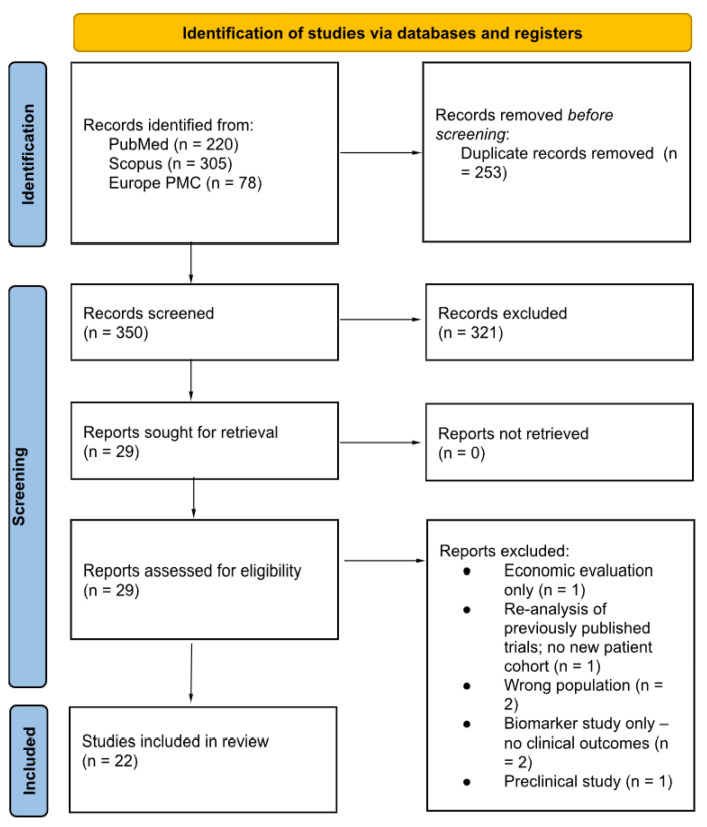
Flow diagram illustrating the identification, screening, eligibility assessment, and inclusion of studies in this review.

The database search yielded 603 unique records. After removing 116 duplicates, 487 titles and abstracts were screened, of which 458 were excluded for not meeting the basic eligibility criteria. Twenty-nine full-text articles were assessed in detail; seven were subsequently excluded. Consequently, 22 studies—13 phase I–III trials, 8 observational cohorts, and 1 case series—met the inclusion criteria and were carried forward into the qualitative synthesis, as depicted in the flow diagram shown in Figure 1.

### 3.2. Study Characteristics

The included studies comprised the following:

- 13 randomized or non-randomized phase I-III clinical trials;
- 8 retrospective cohort or real-world observational studies;
- 1 case-series involving ≥3 patients.

The studies were conducted between 2010 and 2025, covering diverse patient populations with MUM.

### 3.3. Therapeutic Modalities Analyzed

The included studies evaluated one or more of the following immunotherapeutic approaches:

- BTCEs: 15 studies;
- ACTs: including tumor-infiltrating lymphocytes (TILs) (4 studies) and TCR-T cells (0 studies);
- CAR-T and engineered T cells: 0 studies;
- OVs: 3 studies.

A breakdown of outcomes by intervention type is summarized in Table 4, Table 5, Table 6 and Table 7.

### 3.4. Clinical Efficacy

#### 3.4.1. Bispecific T-Cell Engagers

A total of 15 clinical reports encompassing ~1150 HLA-A*02:01-positive patients have explored BTCE therapy in MUM; 14 evaluate the gp100-directed ImmTAC tebentafusp, and 1 the NY-ESO-1–targeted IMCnyeso (López et al. 2025) [30].

The pivotal phase III trial of tebentafusp—analyzed at 12 months (Nathan et al. 2021) [18] and 36 months (Hassel et al. 2023) [12]—raised median overall survival (OS) from 16.9 to 21.6 months (HR 0.68) and lifted 1-year OS from 59% to 73%, despite a modest objective response rate (ORR 5–12%) and median progression-free survival (PFS 2.8–4.6 months).

Early-phase trials and real-world cohorts corroborate these findings, reporting median OS 17–25 months (e.g., Sacco 17.4 mo; Carvajal 25.5 mo) and documenting ORR values up to 23% in selected series. In contrast, IMCnyeso produced nonobjective responses in three evaluable MUM patients, underscoring tebentafusp’s unique clinical activity.

Consistent surrogate markers of benefit have emerged: early treatment-related skin rash (Tomsitz et al. 2024) [25], normal baseline LDH/ALP and higher lymphocyte counts (Sacco et al. 2024) [24], and, most robustly, complete circulating-tumor DNA (ctDNA) clearance by week 12, which corresponded to a median OS of 34.6 vs. 12.7 months (Rodrigues et al. 2024) [19]. A multi-center sequencing study indicates that giving ICI before tebentafusp may further improve outcomes (median OS 33.6 vs. 22.4 months; Dimitriou et al. 2025) [28].

#### 3.4.2. ACTs

##### TILs

Across four prospective reports, TIL therapy was administered to roughly 30 adults with MUM, producing objective response rates that range from 11% (Monberg, TIL + oncolytic adenovirus) to 33% (Chandran, high-dose-IL-2–supported TIL) and giving occasional complete remissions that can persist beyond two years. Responses typically emerge on the first post-infusion scan, involve both hepatic and extrahepatic sites, and, when they occur, often translate into prolonged survival; although, median PFS remains short—about 2–6 months—and median OS in the only study reporting it (Nguyen) was 6.2 months. Efficacy appears to hinge on product quality: Chandran showed higher frequencies of autologous tumor-reactive T cells and greater IFN-γ release in responders, while Murali’s Uveal Melanoma Immunogenomic Score (UMIS) identifies metastases harboring potent but quiescent TILs and predicts benefit after adoptive transfer. Collectively, these data indicate that adoptive-TIL therapy delivers the highest radiographic response rates reported outside BTCEs, though the benefit remains heterogeneous.

##### TCR-T

No data were found regarding TCR-engineered T-cell therapies for MUM that matched our criteria. However, TCR-T represents a next-generation, personalized adoptive-cell transfer strategy that redirects a patient’s own lymphocytes to tumor antigens with high specificity. Because the engineered receptors are restricted to HLA-A*02:01, candidate selection hinges on HLA typing, but, within this subset, TCR-T showed early, meaningful activity in anti-PD-1 refractory melanoma. These modified cells target intracellular melanoma antigens presented on MHC molecules, and the platform is complemented by immune-mobilizing monoclonal TCRs against cancer (immTACs)—CD3-bispecific molecules such as tebentafusp (gp100-directed, already approved for MUM) and emerging PRAME-directed constructs—that recruit bystander T cells to the tumor.

##### CAR-T Therapies

CAR-T therapy for UM is still in its formative stages. Therefore, no data were found regarding CAR-T therapy for MUM that matched our criteria. However, several lines of evidence suggest it could evolve into a meaningful option for this historically treatment-refractory disease. Building on lessons from hematologic malignancies, investigators are now tackling solid-tumor obstacles, such as on-target/off-tumor toxicity, the immune-desert microenvironment, and manufacturing scalability. Combination strategies, particularly pairing OVs with CAR-T cells to inflame UM lesions and improve T-cell trafficking, are being explored, while efforts to mine tumor-specific neoantigens and non-self epitopes continue. Together, these approaches position investigational CAR-T therapies as a potentially transformative addition to the therapeutic arsenal for patients with MUM.

#### 3.4.3. OVs

Across three early-phase trials, ≈29 MUM patients have received OV therapy. No ORR were recorded (aggregate ORR 0%): stable disease (SD) occurred in 4 out of 12, 3 out of 11, and 7 out of 11 evaluable patients, respectively. Despite the absence of radiographic tumor shrinkage, biological activity was evident; Smith reported dose-dependent Tyrosinase Related Protein 1 (TYRP1)-specific T-cell expansion and epitope spreading, with two patients subsequently deriving durable benefit from immune-checkpoint blockade. García observed that the uveal subset lived 3.7-fold longer than matched CM controls after ICOVIR-5 (median follow-up 26 days). These data suggest that current OVs are immunologically active, but clinically weak as monotherapy in MUM. To turn the immune boost that these viruses provide into real tumor shrinkage that shows up on scans, we will probably need to use them in combinations like V937 plus ipilimumab and pick patients with the right biomarkers.

### 3.5. Safety Outcomes

Across the 22 studies analyzed, three consistent toxicity patterns emerged. Tebentafusp generated mechanism-based grade ≥ 3 events in roughly 40–70% of patients—43% in the phase I/II weekly-dosing trial and 71% in the step-up phase I study—yet permanent discontinuation is rare (≈2%) and no treatment-related deaths were reported; toxicities (rash, hypotension, transient transaminase rises, and low-grade cytokine-release syndrome (CRS)) peaked during the first three infusions and waned thereafter, as confirmed by the long-term follow-up cohort where late grade 3–4 events fell to 7%. Adoptive-TIL therapy inevitably produced short-lived grade 3–4 cytopenias from lymphodepletive chemotherapy; additional high-dose i.v. IL-2 toxicities accounted for the single treatment-related death observed (sepsis in Chandran 2017 [34]). When low-dose s.c. IL-2 or virus/DC priming was used, the number of non-hematologic grade ≥ 3 events dropped below 10% (Nguyen 2019 [33]; Monberg 2025 [31]), although blood counts still transiently reached grade 3–4 in every patient.

OVs were generally mild: flu-like symptoms and transient transaminase elevations dominated; scattered grade 3 events kept the serious-AE rate under 20%, and no treatment-related deaths have been reported.

## 4. Discussion

This review provides a comprehensive synthesis of the current landscape of non-checkpoint immunotherapies in MUM, with a focus on BTCEs. In the discussion, each included study was described, and the clinical gaps were complemented with data from preclinical research.

### 4.1. BTCEs

Tebentafusp is a novel bispecific immunotherapeutic agent that represents a significant advancement in the treatment of MUM. It is the first systemic therapy to demonstrate a statistically significant improvement in OS in this patient group. It consists of an affinity-enhanced TCR that is specific to gp100 and is fused to an anti-CD3 effector domain. This enables the redirection of T cells towards melanoma cells that express gp100. The therapy is approved for use in adult patients who are HLA-A*02:01-positive and present with unresectable melanoma or MUM [8,9,10]. The following section summarizes key efficacy and safety data from available studies.

Middleton et al. [21] conducted a multi-center phase I/II study to examine the safety, efficacy, and mechanism of action of tebentafusp. The study included 84 patients with advanced melanoma of the uveal membrane of the eye and the skin. Tebentafusp was generally well tolerated and demonstrated clinical activity in both cohorts, achieving a one-year OS rate of 65%. Biomarker analysis revealed increased serum levels of CXCL10 and decreased circulating levels of CD8+ CXCR3+ T cells during treatment. These findings are consistent with increased tumor infiltration by cytotoxic T cells and are consistent with the results of cytotoxic T-cell redistribution. These results suggest the redirection and movement of T cells into the tumor microenvironment (TME). Additionally, the development of rash and higher serum CXCL10 levels were positively correlated with improved survival. These data support the mechanism of tebentafusp-mediated immune activation by targeting gp100, suggesting its potential therapeutic application in melanoma treatment.

In an international, open-label, phase I/II study, Carvajal et al. [22] examined the effects of tebentafusp in a group of patients who were positive for HLA-A*02:01. A total of 127 patients were enrolled in the phase II trial and received weekly intravenous tebentafusp. The median age was 61 years, and 96% of the patients had liver metastases. Most patients (58%) had elevated baseline lactate dehydrogenase (LDH) levels, and 34% had received ≥2 prior lines of systemic or liver-targeted therapy. Over two-thirds of the patients had previously received immune checkpoint inhibitors, 68% of whom showed primary resistance. The median treatment duration was 5.5 months, and 17% of patients were still receiving treatment at the time the data were collected. The main reason for discontinuing treatment was disease progression (70%). Most of the deaths that occurred during the study were attributed to disease progression; two fell into the “other” category, and no deaths were attributed to the study drug.

Another phase I study by Carvajal et al. [23] was designed to determine the recommended dose of tebentafusp for phase II trials. A three-week gradual dosing regimen was used to evaluate safety, pharmacodynamics, pharmacokinetics, and initial clinical activity in patients with MUM. Forty-two HLA-A*02:01-positive patients with metastatic uveal melanoma who had previously received a median of two treatments were enrolled in the study. Dose escalation identified 68 μg as the recommended phase II dose (RP2D). Dose-limiting toxicity (DLT) occurred at 73 μg and included transient elevation of grade 3–4 aminotransferases. Treatment-related adverse events (AEs) were common, and the ORR was 11.9%. The median PFS was 4.6 months, and the median OS was 25.5 months. One-year OS was 67%. Tumor biopsies showed increased T-cell infiltration after treatment. Serum analyses showed transient induction of pro-inflammatory cytokines, particularly IFN-γ-induced chemokines (CXCL10 and CXCL11). Lower baseline systemic inflammation was associated with longer OS. Early cutaneous toxicity was associated with improved survival, suggesting its potential as a predictive biomarker.

Another phase I/II study by Sacco et al. [24] determined the optimal doses of tebentafusp for patients with previously treated MUM. The study’s primary objective was to determine the overall response rate. A total of 146 patients were included in the study, with a median follow-up of 48.5 months. The majority (91%) received the recommended phase II dose of 68 µg. Despite the modest ORR of 5%, nearly half of the evaluated patients experienced tumor shrinkage, and 66% received treatment after initial progression. The median OS was 17.4 months, and the 1-, 2-, 3-, and 4-year survival rates were 62%, 40%, 23%, and 14%, respectively. Longer OS was associated with female gender, normal baseline LDH and alkaline phosphatase (ALP) values, higher baseline lymphocyte count, and early-onset rash. Circulating-tumor DNA dynamics also correlated with survival; patients with ctDNA deletion by week 9 had significantly better long-term outcomes, even among those with radiographic disease progression. Expression of gp100 did not correlate with OS; however, partial responses were more common in tumors with high gp100 H-scores. A long-term safety analysis showed no new concerns, with most adverse events occurring early and decreasing in severity over time.

In an open-label, phase III clinical trial [12] conducted by Hassel et al., investigators evaluated the long-term efficacy and safety of tebentafusp. In total, 378 patients were randomized at a 2:1 ratio to receive tebentafusp or an investigator-selected therapy (pembrolizumab, ipilimumab, or dacarbazine). After a 36-month follow-up period, the median OS was significantly longer for the tebentafusp group (21.6 months) than for the control group (16.9 months), with an HR for death of 0.68 (95% confidence interval [CI]: 0.54–0.87). The estimated three-year survival rate was 27% for tebentafusp, compared to 18% for the control group. The drug was generally well tolerated, but AEs occurred. The most common were rash (83%), fever (76%), pruritus (70%), and hypotension (38%), and occurred mainly during the early stages of treatment. Only 2% of patients discontinued treatment due to AEs, and there were no treatment-related deaths.

An earlier phase III study created by Nathan et al. [18] also examined the previously described group of 378 patients, where the effects of a one-year intervention with tebentafusp were observed. Also randomized in a 2:1 ratio to receive tebentafusp (*n* = 252) or an investigator-selected therapy (pembrolizumab, ipilimumab, or dacarbazine) (*n* = 126). After 1 year, OS was significantly higher in the tebentafusp group (73%) compared to the control group (59%), with an HR for death of 0.51 (95% CI, 0.37–0.71; *p* < 0.001). Six-month PFS also improved (31% vs. 19%; HR for progression or death, 0.73; 95% CI, 0.58–0.94; *p* = 0.01).

Another phase III study by Rodrigues et al. [19] focused on ctDNA as a potential prognostic and predictive marker in 69 MUM patients treated with tebentafusp, showing the efficacy of the drug in this group of patients. The median PFS was 2.8 months, and the median OS reached 21.8 months. The objective ORR was 10%, with all responses being partial. SD was observed in 34% of patients, while 56% experienced disease progression (PD). ctDNA was detectable at baseline in 61% of patients and was associated with tumor burden indicators, such as LDH levels and size of metastases. Notably, all patients with partial response to tebentafusp had undetectable ctDNA at baseline. Furthermore, ctDNA clearance at 12 weeks post-treatment initiation correlated with significantly improved OS and PFS. Patients with ctDNA clearance had a median OS of 34.6 months, compared to 12.7 months in those without clearance (HR = 7.1, *p* = 0.003). Similarly, median PFS was longer in the clearance group (17.1 vs. 2.6 months, *p* = 0.03). Reduction in ctDNA by ≥90% or ≥50% at 12 weeks was also associated with longer survival outcomes, although results were less statistically robust at the 50% threshold. Importantly, patients with baseline ctDNA positivity who achieved complete clearance at 12 weeks showed survival outcomes comparable to those with undetectable ctDNA from the start, highlighting the potential of ctDNA as a dynamic prognostic and response biomarker in tebentafusp-treated MUM.

Roshardt Prieto et al. [20] conducted a retrospective, single-center study at the University Hospital of Zurich examining 19 HLA-A*02:01-positive patients with MUM who were treated with tebentafusp. Metabolic and morphologic tumor responses were compared using PET Response Evaluation Criteria in Solid Tumors (PERCIST) and Response Criteria in Solid Tumors (RECIST) criteria, respectively. According to RECIST 1.1, the ORR was 10%, with a median PFS of 2.8 months (95% CI: 2.5–8.4) and a median OS of 18.8 months. A comparative analysis of ten patients using both RECIST and PERCIST criteria revealed poor concordance (Cohen’s kappa [κ] ≤ 0), particularly at the initial follow-up (*p* = 0.037). Although the ORR was the same for both criteria, PERCIST identified a longer median PFS (3.1 vs. 2.4 months). Notably, some patients showed a metabolic response without morphologic improvement, suggesting that conventional imaging may underestimate early therapeutic effects. Exploratory analyses revealed that elevated baseline LDH levels and older age predicted a higher risk of progression. Conversely, an early decrease in lymphocyte counts after tebentafusp treatment initiation correlated with a reduced risk of morphologic progression. These results suggest that metabolic imaging may better capture the early effects of tebentafusp treatment in MUM than morphologic criteria alone.

A further noteworthy study is the cohort study by Tomsitz et al. [25], which evaluated the cutaneous side effects frequently caused by tebentafusp. The analysis included 33 patients. Skin lesions were observed in 26 patients (78.8%). The lesions were classified into five categories: (1) symmetrical erythematous patches (83.8%); (2) hemorrhagic macules (11.8%); (3) urticarial lesions (7.4%); (4) blistering lesions (1.5%); and (5) depigmentation of the skin (8.5%) and hair (11.4%). Histopathological analyses revealed lymphocytic dermatitis with the infiltration of CD8+ lymphocytes into the epidermis. This process is related to the infiltration and activation of lymphocytes into drug-stimulated melanocytes. However, it is important to note that this side effect is associated with improved overall patient survival (median: 34 months vs. 4 months, *p* < 0.001) in patients without skin lesions.

Tomsitz et al. [26] also conducted a retrospective multi-center study analyzing the efficacy and safety of tebentafusp in 78 patients with MUM. The median age was 63 years, with an equal number of male and female patients. Most patients had liver metastases (97.4%), and many had previously undergone systemic (51.3%) or local (35.9%) therapies. The study’s results were a median PFS of three months and a median OS of 22 months. Unfortunately, disease progression was observed in 66.2% of patients. In patients who received immunotherapy, the immune checkpoint inhibitors (ICI) before tebentafusp had a median OS of 28 months compared to 24 months for those treated first with tebentafusp (a difference that was not statistically significant). Notably, patients who received the ICI → tebentafusp treatment sequence experienced longer PFS and OS than those who received the reverse sequence. This sequence may be associated with more favorable clinical outcomes. There was no significant difference in OS between patients who received local treatment (e.g., radiotherapy or embolization) and those who did not (22 vs. 24 months, *p* = 0.873).

In another study, the authors focused on the cutaneous side effects of tebentafusp. Staeger et al. [27] describe skin lesions in patients that exhibit characteristics of severe cytotoxic dermatitis, resembling lichen planus and graft-versus-host disease (GVHD). Histopathological examination revealed necrotic keratinocytes, vacuolization of the basal layer of the epidermis, and a dense lymphocytic infiltrate with a predominance of CD3^+^ and CD8^+^ T cells. The presence of numerous CD103^+^ tissue-resident memory (Trm) T cells indicates a long-lasting local immune response. The skin lesions also exhibit endothelial edema and a perivascular inflammatory infiltrate, further resembling acute GVHD. Single-cell RNA sequencing (scRNA-seq) confirmed the presence of activated cytotoxic T cells expressing genes such as GZMB, PRF1, and IFNG, which are indicative of intense inflammation. These data suggest that tebentafusp induces an autoimmune response in the skin by redirecting cytotoxic T cells to skin cells, which may be analogous to the mechanisms observed in GVHD. In this study, there was also an association between the development of cutaneous side effects and longer OS (*p* = 0.0004). However, the occurrence of cutaneous AEs correlated with baseline serum LDH levels, an important prognostic marker.

In a retrospective cohort study, Maurer et al. [14] compared the efficacy and safety of treatment in patients with MUM receiving combination immunotherapy (ipilimumab/nivolumab; ICI) and tebentafusp. The study included 36 patients, with 14 in the ICI group and 22 in the tebentafusp group. The median age of the ICI group was 58 years, and 71% of patients were male. Most had liver and non-liver metastases, primarily in the lungs, lymph nodes, soft tissues, and bone. Thirty-six percent were previously untreated. In the tebentafusp group, the median age was 57 years, 64% of patients were male, and 63% were previously untreated. The most common sites of extrahepatic metastasis were the lymph nodes, soft tissues, lungs, and bones. Elevated serum LDH levels were found in 54% of patients. In the ICI group, the average treatment duration was 77 days, and treatment termination was primarily due to toxicity (50%) and disease progression (43%). The most common side effects were hepatitis, pancreatitis, enteritis, and thyroiditis. In the tebentafusp group, the average treatment duration was 316 days; treatment was terminated in all cases due to disease progression. In the ICI group, an ORR was achieved in 25% of patients, as well as disease control in 58% of patients. The median PFS was 2.9 months, and the median OS was 28.9 months. Elevated LDH levels were associated with shorter PFS and OS. In the tebentafusp group, the ORR was 17%, and the disease control rate (DCR) was 39%. The median PFS was 2.7 months, and the median overall OS was 18.6 months. PFS and OS were longer in patients with a PR or metabolic tumor regression (MTR). Response efficacy in liver metastases correlated with overall treatment efficacy in both groups. In the ICI group, better responses in the liver were associated with longer PFS and OS. This correlation was also noted in the tebentafusp group, although the results were less clear.

Another study by Dimitriou et al. [28] examined the sequence of tebentafusp and ICI, as well as its efficacy and safety, in patients with MUM. The study included 131 HLA-A*02:01-positive patients from 14 centers in eight countries. Group 1 (*n* = 51) consisted of patients who were initially treated with tebentafusp and then, upon disease progression, with ICI. Group 2 (*n* = 80) consisted of patients who were first treated with ICI and then with tebentafusp. The groups were comparable, though they differed in liver lesion incidence and LDH levels. Most patients had not received systemic therapy previously. The most common mutations were GNAQ and GNA11. The median treatment duration with tebentafusp was 24 weeks for Group 1 and 34 weeks for Group 2, and the primary reason for discontinuing treatment was disease progression. By the final follow-up date, 63% of patients had died. The median follow-up was 45.4 and 43.8 months, respectively. Median OS was significantly longer in Group 2 (33.6 months) than in Group 1 (22.4 months, *p* = 0.004). Median PFS was also longer in Group 2 (20.3 months vs. 12 months, *p* = 0.04). Three patients with GNA11/GNAQ mutations (including two with additional BAP1 mutations and central nervous system [CNS] involvement) experienced disease stabilization after treatment with tebentafusp or ICIs. The overall PFS ranged from 14 to 46 months. All patients were alive at the end of follow-up. In Group 1, ICIs yielded modest efficacy after failure of tebentafusp (ORR 4%, DCR 10%). Similarly, in Group 2, the efficacy of tebentafusp was limited after failure of ICIs (ORR 4%, DCR 21%). The treatment sequence ICI → tebentafusp was shown to be associated with longer PFS and OS. However, some factors, such as elevated LDH levels, metastasis in ≥2 organs, and prior systemic treatment, were significantly associated with shorter PFS.

The next study presented was created by Vitek et al. [29] and leans towards the actual efficacy and tolerability of tebentafusp. This is a multi-center retrospective study that enrolled 23 patients from 14 French oncology and oncodermatology centers. The median age was 63 years, and the majority of participants (61%) were women. Fifteen patients had cytogenetic abnormalities or mutations, including monosomy of chromosome 3, polysomy of chromosome 8, and mutations in the BAP1, MBD4, GNAQ, and GNA11 genes. The median time from primary diagnosis to metastatic progression was 3.8 years, and from diagnosis of metastatic disease to initiation of tebentafusp therapy was 9 months. Nearly half of the patients (48%) had previously received at least one line of systemic therapy. At the time of TBP initiation, 83% of patients were ECOG status 0, 32% had elevated alkaline phosphatase levels, and 52% had elevated LDH. All patients were positive for HLA-A02:01. The majority (96%) had multiple metastases: liver only (52%), extrahepatic only (9%), or both liver and extrahepatic (39%). After a mean follow-up period of 12 months, eight patients (35%) passed away. One-year OS was 66%. The median OS was not reached, but exceeded 10.8 months. The response rates were as follows: complete remission, 4%; partial remission, 18%; disease stabilization, 41%; and progression, 36%. The ORR was 23%, and the DCR was 64%. Forty-three percent of patients continued treatment. The median treatment time was 6.9 months. The main reasons for terminating treatment were progression (77%) and death (15%). There was no statistically significant correlation between tebentafusp efficacy and ECOG status, LDH level, AP, metastasis size, or metastasis location (liver only versus extrahepatic).

The last study on bispecific T-cell engagers in this section was the only one to address a different active ingredient than tebentafusp. López et al. [30] investigated IMCnyeso, an immunomobilizing monoclonal T-cell receptor that targets cancer. This agent targets the NY-ESO-1/LAGE-1 isoform A peptide, which is presented by the HLA-A*02:01 tissue compatibility leukocyte antigen. This previous work describes a phase I study involving patients with advanced cancers. The initial study examined 508 patients, including 236 with melanoma and 81 with uveal melanoma. Ultimately, seven patients with melanoma, including three with uveal melanoma, qualified for treatment. The median treatment time was 1.8 months. At least one dose had to be skipped due to side effects in 57% of patients. The most common reason for discontinuing treatment was disease progression (86%). Exposure to the drug increased in a dose-dependent manner. The half-life was approximately 25 h. Immunogenicity was rare; anti-drug antibodies were detected in 2 out of 27 patients, and only one patient experienced pharmacokinetic effects. Dose-dependent increases in cytokine levels and transient decreases in lymphocyte counts were observed. Significant induction of IL-6 and IL-10 (>100-fold) occurred at a dose of 100 μg, accompanied by moderate increases in IFNγ, IL-2, IL-8, and TNF-α. These effects diminished after subsequent doses. Dose-dependent increases in cytokine levels and transient decreases in lymphocyte counts were observed. Significant induction of IL-6 and IL-10 (>100-fold) occurred at a dose of 100 μg, accompanied by moderate increases in IFNγ, IL-2, IL-8, and TNF-α. These effects diminished after subsequent doses. Among a group of seven patients with melanoma, only one patient (14%) achieved disease stabilization; five patients experienced progression, and one patient was unevaluable. The drug showed better results in treating other cancers, such as synovial sarcoma. This finding may warrant further studies of the drug in specific patient groups.

### 4.2. ACTs

#### 4.2.1. TILs

TIL therapy is an innovative form of adoptive-cell immunotherapy. After a small tumor fragment is resected, the resident T-cells are isolated, expanded to billions ex vivo, and reinfused following short lymphodepleting chemotherapy. Because these cells are already primed to recognize tumor-specific antigens, they can target the cancer and eliminate malignant cells while largely sparing healthy tissue. TIL therapy is a promising approach for challenging UM.

In a Monberg et al. study [31], the safety and efficacy of TILs and oncolytic adenovirus TILT-123, producing IL-2 and TNF upon replication, in metastatic melanoma (MM) was tested. The treatment is safe and feasible. Out of 17 enrolled patients, 14 completed therapy by receiving TIL therapy and all six doses of TILT-123. Two patients discontinued treatment due to infection at the injection site or rapid disease progression. One patient did not succeed in TIL expansion, though he completed all virus injections. The ORR after TIL infusion was 2/17 (11.7%). On day 78, the best overall responses (BORs) after TIL infusion are 1 patient with CR with no evidence of disease after 3 years, 1 patient with PR, 4 patients with SD (with 2 patients with UM), and 11 patients with PD, making them ineligible for additional TILT-123 treatment in the extension phase of the trial. Median PFS was 78 days. Importantly, five patients who benefited from the trial are still alive. There were no notable differences in response rates or survival outcomes across the different dose groups.

A Lövgren et al. study [32] focused on evaluating clinical responses and safety in ICI-resistant MM, which was treated with adoptive-cell therapy using TILs combined with autologous dendritic cell (DC) vaccination. The study enrolled 14 patients, 3 of whom had UM. Out of fourteen enrolled patients, ten patients with progressive MM—most of whom had failed prior ICI therapy—were treated with either TIL therapy alone or TILs combined with an autologous DC vaccine. Despite challenges including prior ICI resistance and limited biopsy material, TILs were successfully expanded for all patients, with optimized protocols yielding higher cell numbers in the combination cohort. In the safety/optimization cohort, all patients achieved only mixed responses or SD, but without a durable benefit. In contrast, all four evaluable patients in the combination cohort who received both TILs and DC vaccination demonstrated objective tumor responses: two patients achieved ongoing CRs, one had a durable PR with minimal residual disease, and one experienced a short-lived PR. PET/CT imaging confirmed substantial tumor regression and reduced metabolic activity, highlighting the potential of combining TIL adoptive-cell therapy with DC vaccination to induce meaningful and durable responses in ICI-refractory MM.

The phase II clinical trial from Nguyen et al. [33] determined the efficacy and safety of adoptive-cell therapy with autologous TIL while using a lower, subcutaneous dose of interleukin-2 (IL-2). Therefore, IL-2-related toxicities were reduced when given to patients with unresectable MM. Twelve patients with MM were enrolled in the study, with one patient with ocular melanoma. With the exception of one treatment-naïve participant, every patient in the cohort had already undergone substantial therapy for metastatic disease. Specifically, 10 of the 12 had been exposed to ipilimumab, 9 had previously received PD-1 inhibitors (nivolumab or pembrolizumab), and 2 had tried the ipilimumab–nivolumab combination regimen. In addition, eight patients had also received systemic chemotherapy. Assessing clinical response confirmed two patients achieving PR, one patient with unconfirmed PR (later confirmed as PR), and the rest of the patients had SD for less than 6 months or PD. It was estimated that PFS lasted for a median of 5.1 months, and the estimated median OS was 6.2 months with five deaths during the trial. There were no signs of delayed responses. While just three of the twelve participants achieved objective responses, two others still showed biologic activity from the TIL infusion. In every case, the greatest reduction in target-lesion size appeared on the very first post-therapy scan, followed by subsequent PD.

Chandran et al. investigated in a phase II study [34] the potential of autologous TIL therapy in shrinking MUM. Twenty-seven patients with MUM were enrolled in the study. They had undergone metastasectomy operations to generate therapeutic TIL. Among 27 screened patients, large-scale TIL growth succeeded in 26 (96%), and 21 ultimately received therapy. The remaining two were excluded by interval clinical decline, and three others as well, because of a lack of in vitro tumor reactivity in early cultures. All treated participants completed lymphodepleting cyclophosphamide/fludarabine, then received TILs followed by high-dose IL-2 infusions. After a median follow-up of 7.3 months, objective responses were seen in 7 out of 21 patients (1 complete, 6 partial). Responses lasted 3–20+ months and were ongoing in four cases at last assessment. Two patients achieved durable SD. One treatment-related death occurred from early post-infusion complications before restaging and was therefore unevaluable. Responses consisted of one ongoing CR (CR; 21+ months) and six PRs. Tumor shrinkage was typically evident by the first post-infusion scan and involved both hepatic and extrahepatic lesions. Five of the seven responders had previously failed systemic regimens, including three who had progressed on combined anti-CTLA-4/PD-1 blockade. Notably, one such patient converted from fulminant hepatic progression to a durable CR now approaching two years. Where PRs subsequently relapsed (3–9 months), failure was usually presented as de novo metastases rather than regrowth of regressed deposits. By contrast, in vitro assays of the infused product were strongly predictive: responders received TIL with higher median frequencies of tumor-reactive T cells, greater absolute numbers of such cells, and higher IFN-γ release on autologous tumor challenge. These findings suggest that adoptive TIL therapy can induce rapid and sometimes durable tumor regressions in approximately one-third of heavily pre-treated MUM patients, and that quantitative measures of autologous tumor reactivity may help select effective cell products for future trials.

#### 4.2.2. TCR-T

Although no TCR-T trial has yet reported dedicated clinical-outcome data in a cohort composed exclusively of uveal melanoma patients, TCR-T therapy reprograms a patient’s own lymphocytes with a high-affinity, tumor-specific T-cell receptor, allowing them to recognize intracellular peptides presented on HLA molecules—targets that antibodies cannot reach. Because MUM frequently expresses cancer–testis antigens, such as PRAME or SLC45A2, yet remains immunologically “cold,” TCR-T provides a way to convert antigen expression into a therapeutic weak point. Encouraging pre-clinical work has led to early-phase trials that now test these constructs in patients.

Britten et al. reported a phase I/Ib study of IMA203, a high-affinity TCR-T specific for the PRAME-004 peptide in the context of HLA-A*02:01. Forty-one PRAME-positive patients were treated, only three of whom had metastatic uveal melanoma. Doses from 0.04 to 4.7 × 10^9^ cells m^−2^ were well tolerated; grade ≥ 3 cytokine-release syndrome was rare, and no severe neurotoxicity occurred. The confirmed objective response rate in the overall cohort was 28.9%, with deeper responses correlating with strong PRAME expression and sustained T-cell persistence. Because fewer than five uveal melanoma patients were included, the study did not satisfy our clinical-data inclusion criteria and was therefore excluded from Section 3; nevertheless, it offers proof-of-concept for PRAME-directed TCR-T and justifies larger, dedicated trials in MUM [38].

Rohaan et al. conducted a single-center phase I/IIa dose-escalation study of MART-1-specific TCR-engineered peripheral-blood T cells in twelve patients with metastatic melanoma, including five with MUM. The protocol achieved an overall response rate of 18%, but treatment was halted early because two patients developed grade ≥ 3 cytokine-release syndrome and widespread on-target skin/eye toxicity, underscoring the safety challenges of targeting lineage-shared antigens. Although these data confirm biological activity in MUM, the mixed cohort and pooled read-outs prevented inclusion in our quantitative analysis [39].

A first-in-human phase Ib trial, currently recruiting exclusively MUM patients, has been evaluating hepatic-artery infusion of endogenous CD8^+^ T cells engineered for the melanocyte-restricted antigen SLC45A2 (NCT03068624). Early ASCO reports described efficient manufacturing and favorable pharmacokinetics, but no efficacy or safety endpoints have yet to be released. This study will be the first to provide dedicated clinical read-outs for TCR-T in uveal melanoma once results mature [40]. Pre-clinical evidence continues to justify PRAME and SLC45A2 as preferential targets: PRAME is over-expressed in approximately half of metastatic UMs and can be eradicated by PRAME-specific T cells in vitro [41], while SLC45A2-directed TCRs show potent, highly selective lysis of UM cell lines with negligible off-tumor cross-reactivity [41]. A 2022 narrative review synthesizes these data and highlights combination strategies (e.g., tebentafusp priming) to enhance TCR-T efficacy in the “cold” UM micro-environment [16]. Taken together, the pipeline remains early, with encouraging biological signals but as yet no study meeting our ≥ 3-patient, clinical-outcome threshold for Section 3.

#### 4.2.3. CAR-T—Current Absence of Human Data

CAR-T therapy equips a patient’s own T lymphocytes with a synthetic receptor that recognizes surface antigens independently of HLA, enabling direct lysis of tumor cells. In MUM, the main challenge is finding truly tumor-restricted surface targets—because melanocytic differentiation antigens (e.g., TYRP1, GD2, B7-H3, HER2) are variably expressed on normal eye and skin tissue—yet several pre-clinical programs are beginning to address this gap.

Although no case series involving ≥ 5 MUM patients treated with CAR-T cells has yet been published, recent pre-clinical work is rapidly moving the field toward first-in-human trials. The most advanced construct targets B7-H3, an immunomodulatory B7-family protein that is widely and relatively selectively displayed on uveal melanoma cells while largely absent from healthy tissues. It incorporates inducible caspase-9 (iCas9) as a built-in suicide switch: when exposed to the dimerizing small molecule AP1903, the engineered T cells undergo apoptosis. In a humanized-mouse model, a single infusion of B7-H3 CAR-T cells eradicated extensive liver metastases, and subsequent administration of AP1903 safely ablated the effector population without tumor relapse [17]. Meanwhile, a UCLA group showed that >90% of uveal melanoma tumors express TYRP1; optimized TYRP1-specific CAR-T cells were detected (even at low antigen density), halted the growth of patient-derived xenografts, and caused no off-tumor toxicity, clearing the way for a phase I trial [42]. A complement to these approaches is the “CAR-TIL” strategy: autologous tumor-infiltrating lymphocytes, transiently electroporated with mRNA encoding an anti-HER2 CAR, restored cytotoxicity against UM 92-1 cells even after MHC-I knockout, hinting at a means to forestall antigen-loss escape [43]. A recent translational review further highlights emerging multi-antigen platforms (e.g., GD2-C7R, IL-13Rα2) and notes the first registered protocols actively enrolling UM patients (NCT03635632, NCT04119024) [44]. Taken together, these findings suggest that pairing homogeneously expressed targets (B7-H3, TYRP1) with controllable safety switches (iCas9 + AP1903) and, potentially, regional portal-venous delivery could finally overcome the dual hurdles of safety and tumor heterogeneity, making initial clinical reports feasible within the next few years.

### 4.3. OVs

OV therapy is a novel and promising approach for cancer immunotherapy. Involving either genetically modified or naturally occurring viruses, the therapy allows for the elimination of cancer cells while leaving healthy tissues unaffected. Oncolytic viruses directly kill tumor cells through replication and lysis. A number of clinical studies have investigated the safety profile and preliminary therapeutic potential of the therapy, both as a standalone treatment and in combination with other immune-modulating therapies.

Another phase I study by Smith et al. [35] examined an engineered OV with a Vesicular Stomatitis Virus (VSV) vector modifying expression of interferon-beta and TYRP-1 (VSV-INFβ-TYRP1). The virus was administered both intratumorally into liver metastases and systemically via intravenous infusion. A standard 3 + 3 dose-escalation design was employed, with the primary goal of assessing safety and secondary endpoints including immunogenicity and clinical activity. A total of 12 previously treated MUM patients were enrolled across four dose levels (DL-1-DL-4), with a median follow-up of 19.1 months. Out of all enrolled patients, four achieved SD while eight had PD. Although no objective radiographic responses were observed, immune monitoring revealed dose-dependent T-cell responses to TYRP1, the transgene product, as measured by IFN-γ ELISpot. Importantly, epitope spreading was observed in some patients, with immune responses extending to other melanoma antigens. Two patients of those who exhibited broadened responses and were subsequently treated with ICIs experienced durable clinical benefits.

A phase Ib study by Lutzky et al. [36] evaluated intravenous coxsackievirus A21 (V937) and ipilimumab for patients with MUM who are not HLA-A2:01-positive. Eleven patients with a median age of 65.0 years received a median of 6 injections of V937 and 3.5 infusions of ipilimumab. Only one patient remained on the treatment, with the rest of the patients resigning due to symptomatic disease progression. SD was the best overall outcome, accounting for three patients; the rest of the patients had PD.

A phase I trial by García et al. [37] studied the potential therapeutic usage of oncolytic adenovirus ICOVIR-5 administered intravenously to cutaneous and uveal melanoma patients. Thirteen patients were enrolled in the study, with twelve treated at five dose levels, due to the early progression of one patient. Six patients had UM, and six patients had CM. One patient did not finish the 4-week observation period due to early progression and was later replaced. In terms of efficacy, among the 11 treated patients who underwent at least one disease assessment (day 26), no objective responses (complete or partial) were observed. At lower dose levels, two patients achieved SD. At the highest dose level, SD was noted in five out of six patients. Notably, survival analysis showed that UM patients who did not receive subsequent targeted therapy or anti-PD1 treatment had a 3.7-fold longer survival than CM patients under similar conditions.

Although the following phase I study did not meet our ≥3-patient eligibility threshold for quantitative synthesis, we briefly summarize it here because it provides the only published indication of radiographic response to an oncolytic reovirus in MUM. A phase I study by Comins et al. [45] evaluated the intravenous administration of wild-type reovirus (REOLYSIN) in combination with docetaxel in patients with advanced solid tumors. The study demonstrated good tolerability of the regimen, with no dose-limiting toxicities. Only one patient with UM in the study was admitted, achieving PR. Among 16 evaluable patients, 4 patients achieved PR, and 10 achieved SD. Importantly, the trial confirmed the feasibility of systemic reovirus administration, with viral replication detected in tumor biopsies, supporting its oncolytic activity.

### 4.4. Side Effects

AEs were similar across the studies. Starting from mild and common ones like fever, chills, and rash, finishing at grade 4 hematological issues. All AEs that appeared in the mentioned studies are thoroughly described and analyzed in the paragraph below.

#### 4.4.1. T-Cell Engagers

The long-term safety profile in Hassel et al. [12] remained predictable and manageable, with no new toxicities emerging beyond those described in the primary analysis. The vast majority of patients experienced low-grade, early-onset cutaneous or constitutional effects: rash (83%), pyrexia (76%), pruritus (70%), and hypotension (38%) were most common, peaking during the first four weeks of step-up dosing and tapering in both frequency and intensity thereafter. Grade 3–4 treatment-related events occurred in 47% of patients, driven mainly by severe rash (19%) and isolated laboratory liver-enzyme elevations, such as aspartate aminotransferase (6%). CRS, adjudicated post hoc, was frequent (89%) but mostly mild-to-moderate (grades 1–2 in 88%); grade 3 episodes were rare (1%) and confined to the induction phase. Beyond six months, new high-grade toxicities were uncommon and largely limited to transient biochemical abnormalities coinciding with disease progression. Only 2% of tebentafusp-treated participants discontinued therapy for treatment-related reasons, and no treatment-related deaths were observed. The development of anti-drug and neutralizing antibodies in up to 29% and 19% of patients, respectively, had no discernible impact on either safety or efficacy, underscoring tebentafusp’s acceptable tolerability profile over extended administration.

Across the phase III Nathan et al. trial [18], tebentafusp was associated with a distinct, mostly low-grade toxicity profile characterized by cytokine- and skin-mediated events. Any-grade AEs occurred in nearly all participants, with pyrexia (76%), chills (47%), hypotension (38%), rash (83%), pruritus (69%), and erythema (23%). Grade 3–4 treatment-related AEs (TRAEs) were more frequent with tebentafusp, rarely leading to permanent discontinuation. No treatment-related deaths were reported. CRS emerged in 89% of tebentafusp-treated patients, predominantly grade 1–2 (88%), confined to the first three infusions, and effectively managed with antipyretics, fluids, and/or corticosteroids without mandated prophylaxis. Importantly, AE incidence and severity diminished after the initial 4-week intrapatient dose-escalation phase, enabling outpatient administration from week 4 onward. Development of anti-tebentafusp antibodies was observed in 29% of patients, with no discernible impact on drug exposure, hypersensitivity risk, or overall survival. Collectively, these data indicate that while tebentafusp induces frequent immune-mediated AEs, they are generally early-onset, manageable, and seldom lead to treatment cessation, supporting its tolerability in MUM.

A Rodrigues et al. study [19] focused on assessing ctDNA in patients with MUM. It did not include any data regarding the side effects of the provided treatment.

The Prieto et al. study [20] focused on determining the patterns of radiological response to tebentafusp in patients with MUM. It did not include any data regarding the side effects of the treatment.

In the first-in-human phase I Middleton et al. [21] study, tebentafusp displayed a high overall incidence of TRAEs but a manageable safety profile. Almost all participants (83/84; 99%) experienced at least one TRAE, yet discontinuations for toxicity were rare (2/84; 2.4%). Dose-limiting toxicity occurred only at the 900-ng kg^−1^ weekly dose (two patients), establishing a maximum tolerated dose of 600 ng kg^−1^ and a recommended phase-II dose of 68 µg. Any-grade CRS was observed in 60% of patients, but the vast majority was mild-to-moderate (grades 1–2: 56%; grade 3: 3.6%; no grade 4/5 events). Across both dosing schedules, the most frequent TRAEs were rash (68%), pruritus (70%), pyrexia (57%), periorbital edema (49%), fatigue (54%), nausea (52%), and hypotension (33%). Grade ≥3 toxicity was reported in 43% of patients, driven chiefly by rash (26%) and lymphopenia (13%), with serious hypotension (8%) and pyrexia (5%) occurring less often. Importantly, the safety profile was consistent between the weekly and intensified regimens, and no dose-limiting toxicity emerged at the 600-ng kg^−1^ level, supporting the selected dose for subsequent trials.

In a Carvajal et al. [22] prospective study of 127 HLA-A*02:01-positive patients receiving tebentafusp, every participant experienced at least one TRAE, with toxicity as largely early-onset, reversible, and rarely leading to permanent cessation of therapy. The dominant toxicities, mirroring the drug’s mechanism, were skin events from gp100-positive melanocyte targeting (rash 87% any-grade, 16% grade ≥ 3; pruritus 67%, 4% grade ≥ 3) and cytokine-mediated effects from T-cell activation (pyrexia 80%, chills 64%, nausea 59%). CRS occurred in 86% of patients but was almost exclusively mild-to-moderate (grade 3 = 3.1%; grade 4 = 0.8%), emerged within 8–10 h of dosing, and was largely confined to the first three step-up infusions; management with antipyretics, intravenous fluids, and occasional steroids or tocilizumab proved effective. Overall, 47% of patients reached grade ≥3 toxicity, with grade 4 events in 6% (most commonly hypotension or laboratory abnormalities), only 3% discontinued therapy, and there were no treatment-related deaths. Hospitalizations (20%) and dose interruptions (17%) were infrequent, and the incidence of key toxicities, such as rash, fell from ~65% in weeks 1–3 to 23% by week 8. These findings confirm that, despite a high rate of mechanism-based TRAEs, tebentafusp’s safety profile is predictable, front-loaded, and manageable with standard supportive care.

In the 42-patient cohort Carvajal et al. study [23], TRAEs were universal (100%), and high-grade toxicity was common and largely manageable. In total, 71% of patients experienced grade ≥ 3 TRAEs, driven primarily by abdominal pain (12%), hypotension (9%), fatigue (9%), and hypophosphatemia (9%). Serious adverse events occurred in 38% of participants—most often abdominal pain, transient transaminase elevations (AST 7%, ALT 5%), and hypotension and hyperbilirubinemia (each 5%)—but led to permanent drug discontinuation in only two cases (CRS and abdominal pain). CRS was reported in 90% of patients, almost exclusively grade 1–2 (98%); a single grade 4 episode (2%) resolved with supportive care but met protocol criteria for discontinuation. Skin toxicities, an on-target reaction to gp100-expressing melanocytes, were frequent—rash and pruritus, each 83%, dry skin 64%, pigment or erythema changes 57%—yet two-thirds were grade 1–2, presented within the first few doses, and responded to antihistamines or topical corticosteroids, with no cases of Stevens–Johnson syndrome or toxic epidermal necrolysis. Increases in transaminase (21%) were generally early, CRS-associated, and self-limiting, whereas persistent elevations correlated with disease progression. Notably, AE incidence and severity (rash, hypotension, pruritus, and pyrexia) declined on continued dosing, no anti-IL-6 therapy was required, and no treatment-related deaths occurred, underscoring a predictable, front-loaded but controllable safety profile for tebentafusp.

In the Sacco et al. study [24], long-term follow-up confirmed that tebentafusp’s toxicity profile is front-loaded and attenuates with ongoing therapy. All four treatment-related discontinuations arose within the first treatment cycle, and no additional safety signals emerged thereafter. Beyond 12 months on the drug, only seven grade 3–4 events were recorded in three patients (7%), all coinciding with tumor progression and largely confined to laboratory abnormalities. Clinically manifested toxicities that are prominent early—rash, pyrexia, hypotension, and transient liver-function-test elevations—became infrequent after six months, with no late-grade 3–4 rash and virtually no CRS manifestations beyond week 8. Taken together, these data indicate that prolonged tebentafusp exposure is not associated with cumulative or delayed organ toxicity and that the risk of severe adverse events diminishes markedly after the initial dose-escalation period.

In the Tomsitz et al. prospective dermatologic assessment of 105 tebentafusp infusions [25], acute cutaneous eruptions were documented after 68 administrations (64.7%), invariably emerging after the first dose and involving chiefly the face (69%) and torso (68%), with lesser involvement of upper limbs (37%), neck (19%), lower limbs (4%), and genital skin (3%). Pruritus accompanied 47% of reactions. Onset was typically rapid—a median 6.5 h (range 3.5–7 h) in recorded cases—and intensity declined with subsequent doses, decreasing in 85% of instances by the third infusion; four patients reported self-limiting recurrences that eventually abated. Clinical phenotypes were heterogeneous: symmetrical erythematous patches predominated (83.8%), followed by distal hemorrhagic macules (11.8%), urticarial plaques (7.4%), and rare bullous detachment (1.5%). All eruptions, except blistering lesions, resolved without sequelae before the next weekly dose, and none prompted treatment discontinuation; symptomatic cases were managed successfully with topical corticosteroids. Late, permanent pigmentary changes were also observed: vitiligo-like depigmented macules in 8.5% and hair depigmentation in 11.4%, appearing 8–19 weeks after therapy initiation and reflecting on-target melanocyte loss.

In the Tomsitz et al. [26] retrospective multi-center study, acute toxicity was frequent but mostly low-grade and manageable. Within the first 24 h post-infusion, 88.5% of patients developed TRAEs, driven chiefly by CRS in 71.2%. CRS was grade 1 in two-thirds of cases (pyrexia ± chills only), grade 2 in 28.6% (additional hypotension responsive to i.v. fluids), and grade 3 in 5.4% (hypotension requiring corticosteroids or tocilizumab). No grade 4 events occurred. Skin reactions arose in 53.8%, were grade 1–2 in >95%, and responded to topical corticosteroids and antihistamines. A single grade 3 bullous eruption was reported. Early non-skin, non-CRS toxicities were uncommon (nausea/vomiting 14%, abdominal pain 3%), and one patient experienced tumor-lysis syndrome after the first dose. Premedication, principally antihistamines and antipyretics, was used in one-third of patients to mitigate TRAEs. Late on-target pigmentary effects included vitiligo-like macules (11.5%) and leukotrichia (5.1%). Only three patients (3.8%) discontinued treatment because of toxicity, and no treatment-related deaths occurred, underscoring a largely controllable safety profile concentrated in the first day following infusion.

In the phase III Staeger et al. trial [27], cutaneous AEs were almost universal and predominantly mild, reflecting the drug’s on-target engagement of gp100-expressing melanocytes rather than off-target toxicity. “Rash”, a composite category encompassing erythematous, maculopapular, and vesicular eruptions, occurred in 83% of participants, while pruritus and pigmentary changes were reported in 69% and 45%, respectively. These cutaneous AEs emerged very early after treatment initiation, rarely exceeded grade 1–2 severity, and never prompted permanent drug discontinuation. Their predictable timing and benign course suggest that sequential skin biopsies could serve as an accessible pharmacodynamic window into tebentafusp’s mechanism, allowing for real-time interrogation of T-cell activation, on-target melanocyte damage, and the contribution of bystander immune cells.

In Maurer et al. [14], among five tebentafusp-treated patients who underwent paired liver-metastasis biopsies, histopathology revealed heterogeneous intralesional immune activity that only partly paralleled imaging findings. The two patients whose liver lesions progressed radiologically displayed no pathological tumor regression and only sparse, isolated CD8^+^ T-cell infiltrates. By contrast, two other patients showed dense intratumoral and peritumoral lymphocytic aggregates, predominantly CD8^+^ T cells, despite radiological impressions of either mixed stability/progression. Notably, one patient exhibited a marked pathological response, with <10% viable tumor tissue accompanied by necrosis, fibrosis, and macrophage-rich inflammation. These observations suggest that tebentafusp can elicit robust intratumoral immune infiltration and occult tumor necrosis that may not be fully captured by conventional imaging in uveal melanoma liver metastases.

In the Dimitriou et al. [28] retrospective two-cohort analysis, immunotherapy-related toxicity profiles diverged markedly between ipilimumab + nivolumab and tebentafusp. Among patients receiving dual-checkpoint blockade, only one in four completed all four induction doses. TRAEs were recorded in 51% of group 1 versus 66% of group 2; high-grade (≥grade 3) events occurred in 14% and 29%, respectively. Rash, hepatitis, colitis, and thyroiditis predominated, with colitis the leading grade ≥ 3 toxicity (6% and 14%). Tebentafusp was administered for a far longer median duration (≈20–21 infusions) and produced TRAEs in 96% of group 1 and 88% of group 2, chiefly cytokine- or skin-mediated. CRS was more frequent in pre-treated patients (27% vs. 15%), while rash (39% vs. 43%) and fever (5% vs. 6%) were common across cohorts. Severe TRAEs were similar or lower than with checkpoint blockade (22% vs. 15%), driven mainly by transient liver-function abnormalities (10% and 8%) and rash (8% and 3%). Importantly, no grade 3/4 CRS, immune effector-cell neurotoxicity, or treatment-related deaths were reported with tebentafusp, underscoring its predominantly manageable, mechanism-based safety profile relative to the broader autoimmune toxicities of combined CTLA-4/PD-1 inhibition.

In the Vitek et al. cohort study [29], tebentafusp produced a predictable but largely manageable toxicity profile: 87% of the 23 evaluable patients reported at least one TRAE, driven mainly by cytokine-mediated symptoms, fever (83%), chills (73%), and hypotension (29%), and on-target skin effects such as pruritus (70%) and rash (61%). Severe (grade ≥ 3) toxicity was limited to 30% of patients, almost exclusively rash (five cases) and fever (four cases), with no grade ≥ 3 hypotension or hypoxemia and no treatment-related discontinuations or deaths. CRS interventions followed standard recommendations (antipyretics, antihistamines, corticosteroids, oxygen, or volume support as needed), and no grade 3–4 CRS occurred beyond the step-up phase. AEs clustered during early dose escalation, declining thereafter; 45% of affected patients were symptom-free by week 3. Late, low-grade pigmentary changes (vitiligo-like depigmentation, poliosis) and palmar–plantar erythrodysesthesia were infrequent, and a single case of acute myeloid leukemia 55 days after therapy initiation was reported without established causality. Overall, tebentafusp’s side-effect spectrum remained front-loaded, immune-mediated, and readily controlled with supportive care.

In the phase I dose-escalation study by López et al. [30], TRAEs were common yet largely mild-to-moderate and reversible. The predominant any-grade toxicities (≥20% of the 28 participants) were cytokine-mediated or flu-like, pyrexia (79%), headache (61%), and chills (46%), along with CRS in 43% of patients. All CRS episodes were grade 1–2, arose after doses ≥ 30 mg in the absence of pre-medication, occurred within the first few infusions, and resolved the same day with symptomatic care, occasionally requiring tocilizumab or corticosteroids. Incidence and severity of TRAEs declined markedly after the first three doses, and no CRS was seen beyond week 6. Grade 3–4 toxicities were infrequent, led by neutropenia (14%) and lymphopenia (11%), emerging 4–6 weeks after initiating therapy at doses ≥ 100 mg; counts normalized within two weeks after brief dose interruptions and, when needed, growth-factor support. Two dose-limiting toxicities were recorded: grade 3 febrile neutropenia and a transient grade 4 transaminase rise, neither precluded continued treatment at the target dose. Crucially, there were no treatment-related deaths, discontinuations, or neurotoxicity events, underscoring a manageable safety profile.

#### 4.4.2. TILs

In a Monberg et al. study [31], the administration of TILT-123 and subsequent TIL therapy was generally well tolerated. The most common treatment-related AEs following TILT-123 injections were fever (53%), fatigue (24%), and nausea (24%), with most events being mild (grade 1–2) and resolving spontaneously or with standard care. All AEs experienced before day 36 were easily treated or resolved spontaneously, except for one patient with dry skin and one patient with nausea. One patient experienced grade 3 fever after TILT-123, and another had injection site pain of grade 3 severity; both resolved within days. No dose-limiting toxicities were reported. One patient developed a bacterial infection at the injection site, which was resolved with antibiotics. After receiving both TILT-123 and TILs, the most frequent AEs remained as before: fever (65%), fatigue (29%), and nausea (29%). TIL infusions produced expected effects, including fever (47%) and chills (35%). One patient with pre-existing adrenal insufficiency developed grade 4 adrenal crisis after TIL therapy but recovered fully with prompt corticosteroid management. Overall, grade 3–4 AEs were infrequent, did not increase with dose escalation, and serious adverse events were manageable, with a total of seven SAEs reported across both treatments.

In a Lövgren et al. study [32], lymphodepleting cyclophosphamide/fludarabine was administered to all ten treated patients, with two receiving dose reductions for mild renal impairment; expected cytopenias were universal, and one patient required autologous stem-cell rescue for prolonged marrow suppression. Toxicity following TIL infusion was generally mild-to-moderate (fever, chills), although one patient developed a capillary-leak-syndrome-like reaction that led to withholding interleukin-2 (IL-2). IL-2 support produced the most severe adverse events, including hypotension, fluid overload, electrolyte disturbances, and high-grade fevers; low-dose intravenous hydrocortisone was used to blunt emerging cytokine-release features. Only four of five patients in the safety cohort completed all fourteen planned IL-2 doses, and none in the combination cohort did so, with most discontinuations being triggered by capillary leak syndrome. One treatment-related death occurred: a patient with baseline hydronephrosis tolerated chemotherapy and TIL infusion but deteriorated two days after the tenth IL-2 dose, dying of refractory metabolic acidosis with no clear anatomic cause at autopsy. The autologous dendritic-cell vaccine itself was well tolerated, with no local or systemic toxicity reported. Notably, three responding patients developed vitiligo, consistent with on-target autoimmunity and heightened systemic antitumor immunity.

Across the 12-patient cohort in the Nguyen et al. phase II clinical trial [33], treatment was not associated with any grade 5 events, but dose-limiting toxicities were frequent and predictable. All participants developed grade 3–4 cytopenias after cyclophosphamide/fludarabine lymphodepletion. Ten patients recovered with standard transfusion support plus G-CSF, whereas two required rescues with autologous stem-cell infusion for marrow aplasia or delayed engraftment. Febrile neutropenia occurred in seven patients, with only one experiencing culture-positive, multi-organ infections. Low-dose IL-2 was generally well tolerated: eight patients completed ≥7 out of 9 planned injections, and most IL-2-related events (vascular-leak manifestations, such as peripheral/pulmonary edema, transient hypotension or creatinine rise, and constitutional symptoms) were grade 1–2 and managed in the ward with supportive care or dose omission. Three participants stopped IL-2 after four doses because of grade 2–3 edema or rash; one of them later developed an immune-reconstitution pneumonitis necessitating brief mechanical ventilation. Non-hematologic grade 3–4 events attributable to chemotherapy or TIL infusion were uncommon but included electrolyte disturbances and two self-limited episodes of post-infusion hypoxia that postponed IL-2 administration. Overall, the adverse-event profile was dominated by reversible hematological toxicity typical of preparative chemotherapy, with most other side effects manageable without intensive care, supporting the feasibility of outpatient administration in appropriately selected patients.

The Chandran et al. phase II study [34] was generally well tolerated, with toxicities largely attributable to the preparative lymphodepleting regimen rather than to the TIL infusion itself. Predictably, every patient developed transient grade ≥ 3 hematologic toxicities: lymphopenia, neutropenia, and thrombocytopenia, with grade 3 anemia in 67%; these abnormalities resolved within 7–10 days as immune reconstitution occurred. Febrile neutropenia and culture-documented infections were each observed in 29% of patients, and, although most were low-grade and responsive to supportive care, one patient with pre-existing emphysema succumbed to sepsis-induced multiorgan failure, representing the sole treatment-related death. Non-hematologic adverse events were infrequent and generally mild, including transient renal (creatinine elevation, 29%) and hepatic laboratory abnormalities (AST or bilirubin elevations, 14%), dyspnea (19%), and fatigue (19%). Notable serious events comprised a catheter-associated venous thrombosis that resolved with thrombolysis, grade 4 cerebrovascular ischemia accompanying the fatal sepsis, and a delayed grade 4 fludarabine-related motor neuropathy that improved with rehabilitation. Autoimmune toxicity was rare, limited to one case of patchy vitiligo, and no acute infusion toxicities were seen. Overall, the safety profile underscores that, aside from the anticipated short-lived cytopenias, severe or irreversible toxicities were uncommon.

#### 4.4.3. OVs

A treatment evaluated in the Comins et al. [45] study was well tolerated. The most common side effects were mild and included flu-like symptoms (fever, chills, headache), diarrhea, fatigue, and neutropenia. Most severe toxicities (grade 3–4) were limited: there were six grade 4 events in total, all but two were episodes of neutropenia, mostly linked to prior docetaxel use and previous extensive chemotherapy. One patient developed sepsis but recovered and completed treatment. One patient with hepatocellular carcinoma experienced a grade 3 increase in aspartate aminotransferase levels on the fifth day of the first treatment cycle, leading to a pause in therapy. As a result, this patient was replaced within the study group. Flu-like symptoms typically appeared 2–4 days after reovirus administration, were more pronounced during the first cycle, and were easily managed with paracetamol and nonsteroidal anti-inflammatory drugs (NSAIDs). The severity of neutropenia was related more to prior chemotherapy than to the reovirus dose. Overall, severe toxicities were uncommon and manageable.

For AEs in the Smith et al. study [35], across dose levels (DLs) 1–2, common side effects included fatigue, grade 1–2 CRS, and low blood counts (decreased platelets, total white blood cells, lymphocytes, neutrophils). One patient at DL2 developed a VSV infection with prolonged flu-like and gastrointestinal symptoms. At DL3, frequent side effects were grade 1 CRS, low platelets and hemoglobin, and elevated liver enzymes (AST, ALT). At DL4, side effects included fatigue, fever, grade 2–3 CRS, low platelets and lymphocytes, and raised AST. Overall, side effects did not notably worsen with higher doses. One patient at DL4 had dose-limiting toxicities (grade 3 low platelets, grade 3 elevated AST, and grade 3 CRS due to hepatitis). Three patients were treated at DL4 with careful monitoring before enrolling the next. AEs did not significantly increase with dose escalation.

For the phase Ib study by Lutzky et al. [36], no dose-limiting toxicities (DLTs) occurred. Most patients (91%) experienced treatment-related side effects, mainly mild-to-moderate diarrhea, fatigue, muscle pain, joint pain, chills, nausea, and itching. No grade 4 or 5 treatment-related side effects were seen. Two patients (18%) had grade 3 diarrhea linked to ipilimumab; none were linked to V937. Serious side effects occurred in four patients (36%), including grade 3 acute myocardial infection, fever, and hydronephrosis in one patient. Grade 3 bile duct blockage, brain swelling, and colitis were all observed in only one patient, with colitis likely related to ipilimumab. Some treatments were delayed or paused due to these events. Two patients (18%) stopped treatment due to diarrhea or chest pain. Five patients (45%) died during the study, but none of the deaths were attributed to the treatment.

In a phase I trial by Garcia et al. [37], no significant toxicities were observed at dose levels 1a–3a. At level 4a, one unrelated grade 2 asthenia did not limit dose escalation. Acute toxicities mainly included a flu-like syndrome (fever, chills, myalgia/arthralgia, headache, nausea, vomiting, and diarrhea) occurring within 4–6 h post-infusion and lasting 2–4 days. At dose level 5a, the first patient experienced grade 3 transaminitis and grade 3 thrombocytopenia, which resolved within days. A second patient at this level developed grade 3 liver toxicity (AST elevation) that resolved by day 12. Both patients had normal baseline liver function; only one had liver metastases. As a result, dose level 5a was declared the maximum tolerated dose (MTD), and the recommended phase II dose was set at the lower level, 4a. Other notable toxicities at level 5a included grade 2 neutropenia and grade 2 AST elevation. Overall, no grade 4 nonhematological toxicities were reported.

Pre-clinical work has begun to delineate how oncolytic viruses might be rationally deployed against uveal melanoma. An HSV-1 vector armed with GM-CSF produced sub-micromolar IC50 values in 92-1, MUM2B, and MP41 cells and shrank orthotopic xenografts by >70%, prolonging survival while triggering F4/80^+^ macrophage recruitment [46]. Leveraging innate signaling, the same backbone combined with the TLR-3 agonist poly(I:C) re-activated NF-κB in tumor cells and tumor-associated macrophages; co-treatment amplified virus-mediated cytotoxicity and promoted M1 polarization beyond that seen with either agent alone [47]. A complementary “viro-chemo” strategy pairs the E1B-deleted adenovirus H101 with the alkylating agent dacarbazine: in vitro, the combination reduced UM cell viability by 60–70% versus 30–35% for monotherapies, drove G2/M arrest, and spared retinal pigment epithelial cells [48].

### 4.5. Emerging Multimodal Immunotherapy Strategies and Options for HLA-A*02:01-Negative Patients

Although the present review focuses on non-checkpoint immunotherapies, such as bispecific T-cell engagers, adoptive-cell transfer, and oncolytic viruses, a parallel line of development involves multimodal strategies that combine immunotherapy with liver-directed or targeted therapies. These approaches are particularly relevant for patients with metastatic uveal melanoma who are ineligible for tebentafusp because they lack the HLA-A*02:01 allele, as well as for those who recurrence after BTCE-based treatment. Hanratty et al. [49] recently reviewed several multimodal strategies, highlighting combinations of immunotherapies with other systemic agents and liver-directed therapies. Retrospective analyses of ICIs combined with transarterial chemoembolization (TACE) and surgical resection have shown a significant improvement in overall survival compared with historical controls. In addition, several specific therapies are currently being evaluated in combination with ICIs, including hepatic perfusion with melphalan, selective internal radiotherapy (SIRT), TACE, and immunoembolization. Early clinical data have shown encouraging disease control and suggest a potential improvement in overall survival. Importantly, they also discussed emerging targeted therapies specifically focused on HLA-A*02:01-negative MUM, such as the phase II/III clinical trial evaluating a combination of protein kinase C inhibitor (darovasertib) with a mesenchymal–epithelial transition factor (MET) inhibitor (crizotinib) as first-line treatment in this population [50,51,52]. In parallel, the phase II PLUME trial of pembrolizumab plus lenvatinib suggests that rational combinations of checkpoint blockade and tyrosine kinase inhibition may yield clinically meaningful activity both in HLA-A*02:01-negative patients and in HLA-A*02:01-positive patients treated with tebentafusp in the past. The study met the predefined statistical success criteria in both cohorts, while maintaining a manageable safety profile [53]. However, these findings should be interpreted with caution, given the monocentric setting and limited sample size. Future larger, multi-center randomized trials are required to confirm efficacy and define optimal patient selection.

Overall, systemic treatment options for HLA-A*02:01-negative patients remain limited, underscoring the need for further studies to develop new therapeutic strategies.

## 5. Conclusions

Tebentafusp has definitively shifted the therapeutic landscape of metastatic uveal melanoma by delivering the first reproducible overall-survival advantage, thereby establishing bispecific T-cell engagement as the current standard for HLA-A02:01-positive patients; nevertheless, its modest radiographic response rate and dependence on a single HLA allele underscore an urgent need for complementary strategies. Based on the 22 studies included in this review, the adoptive transfer of tumor-infiltrating lymphocytes demonstrates the highest potential for deep, durable remissions but remains constrained by logistical complexity, toxicity of preparative regimens, and the limited scale of prospective data, positioning it as an investigational option best pursued within centers experienced in cell therapy. Oncolytic viruses, while safe and immunologically active, have yet to translate biological effects into objective tumor control, suggesting their greatest value may emerge in combination regimens that prime or amplify subsequent immune interventions. Collectively, these findings argue for biomarker-driven trials that rationally pair BTCEs, ACTs, OVs, and checkpoint inhibitors, prioritize the inclusion of HLA-A02:01-negative populations, and incorporate dynamic markers, such as early skin toxicity or circulating-tumor-DNA kinetics, to refine patient selection and sequencing, with the ultimate goal of transforming the survival plateau in this historically recalcitrant disease.

## Figures and Tables

**Table 1:** The complete search protocols and results for each database.

Database	Search String	Date Searched	Results Returned
PubMed	(“uveal melanoma” OR “ocular melanoma” OR …)	31 May 2025	220
Scopus	[string adapted to Scopus syntax]	31 May 2025	305
Europe PMC	(((((“uveal melanoma” OR “ocular melanoma” OR …)	31 May 2025	78

**Table 2:** Inclusion and exclusion keywords.

Inclusion Keywords	Exclusion Keywords
uveal melanoma	murine model
ocular melanoma	mouse model
choroidal melanoma	animal study
iris melanoma	xenograft
tebentafusp	preclinical
ImmTAC	review article
PRAME	case report
tumor-infiltrating lymphocyte	editorial
TIL	in vitro
lifileucel	protocol
adoptive-cell therapy	opinion
CAR-T	meta-analysis
CAR T	systematic review
TCR therapy	rats
T-cell receptor therapy	syngeneic
oncolytic virus	orthotopic model
natural killer cell therapy	

**Table 3:** Characteristics of the studies included in this narrative review that evaluated BTCEs, cell-based therapies (TIL, CAR-T, and TCR-T), and other non-checkpoint immunotherapies in adults with MUM (2010–2025).

Author (Year)	Country	Type	N	Intervention	DOI
Hassel et al. (2023) [12]	Multi-center: Germany, France, USA, UK	Randomized controlled trial, phase III	378	Tebentafusp	10.1056/NEJMoa2304753
Nathan et al. (2021) [18]	Multi-center: UK, USA, Australia, EU	Clinical trial, phase III	378	Tebentafusp	10.1056/NEJMoa2103485
Rodrigues et al. (2024) [19]	France	Prospective cohort study	69	Tebentafusp	10.1038/s41467-024-53145-0.
Roshardt Prieto et al. (2024) [20]	Switzerland	Retrospective cohort study	19	Tebentafusp	10.1097/CMR.0000000000000952
Middleton et al. (2020) [21]	UK and USA	Clinical trial, phase I/II	84	Tebentafusp	10.1158/1078-0432.CCR-20-1247
Carvajal et al. (2022a) [22]	Multi-center: USA, UK, Germany, Canada, Spain	Clinical trial, phase I/II	127	Tebentafusp	10.1038/s41591-022-02015-7
Carvajal et al. (2022b) [23]	USA and UK	Clinical trial, phase I	42	Tebentafusp	10.1200/JCO.21.01805
Sacco et al. (2024) [24]	Multi-center: UK, USA, Canada, Spain, Germany	Clinical trial, phase I/II	146	Tebentafusp	10.1136/jitc-2024-009028
Tomsitz et al. (2024) [25]	Germany	Prospective single-center cohort	33	Tebentafusp	10.1016/j.jaad.2024.08.037
Tomsitz et al. (2023) [26]	Germany and Switzerland	Retrospective multi-center cohort	78	Tebentafusp	10.3390/cancers15133430
Staeger et al. (2025) [27]	Switzerland	Prospective biomarker cohort	11	Tebentafusp	10.1172/JCI181464
Maurer et al. (2024) [14]	Switzerland	Retrospective cohort	22	Tebentafusp	10.1007/s10238-024-01497-8
Dimitriou et al. (2025) [28]	14 centers, 8 countries	Retrospective multi-center sequencing study	131	Tebentafusp	10.1016/j.ejca.2024.115161
Vitek et al. (2024) [29]	France	Retrospective multi-center cohort	23	Tebentafusp	10.2340/actadv.v104.41297
López et al. (2025) [30]	UK, USA, Canada, EU	Phase I, first-in-human dose-escalation	3	NY-ESO ImmTAC	10.1016/j.xcrm.2025.101994
Monberg et al. (2025) [31]	Denmark, France, Finland	Clinical trial, phase I	17	TIL therapy	doi.org/10.1016/j.xcrm.2025.102016
Lövgren et al. (2020) [32]	Sweden	Open-label, single-center, two-cohort phase I trial	10	TIL therapy	10.1080/2162402x.2020.1792058
Nguyen et al. (2019) [33]	Canada	Clinical trial, phase II	12	TIL therapy	10.1007/s00262-019-02307-x
Chandran et al. (2017) [34]	USA	Clinical trial, phase II	21	TIL therapy	10.1016/s1470-2045(17)30251-6
Smith et al. (2023) [35]	USA	Dose-escalation safety trial, phase I	12	Oncolytic VSV	10.3389/fimmu.2023.1279387
Lutzky et al. (2023) [36]	USA	Multi-center trial, phase Ib	11	Oncolytic CVA21	10.1007/s00432-022-04510-3
García et al. (2019) [37]	Spain	Dose-escalation, phase I study	12	Oncolytic adenovirus	10.1089/hum.2018.107

**Table 4:** BTCEs in UM results.

Study	Phase	Intervention Type	N	Key Outcomes Reported	Selected Remarks
Middleton et al. (2020) [21]	Clinical trial, phase I/II	Tebentafusp—safety, efficacy, and mechanism of action	84	1-year OS: 65%	Increased CXCL10, CD8+, and CXCR3+ T-cell redistribution; rash linked to better OS
Carvajal et al. (2022) [22]	Clinical trial, phase I/II	Tebentafusp	127	Median OS: 16.8 mo, main discontinuation due to progression (70%)	96% liver mets; 68% had prior ICI resistance; 17% on treatment at data cut-off
Carvajal et al. (2022) [23]	Clinical trial, phase I	Tebentafusp	42	ORR: 11.9%, PFS: 4.6 mo, OS: 25.5 mo, 1-year OS: 67%	RP2D: 68 µg; CXCL10/11, IFN-γ upregulated; rash predictive of OS
Sacco et al. (2024) [24]	Clinical trial, phase I/II	Tebentafusp	146	ORR: 5%, OS: 17.4 mo; 1-, 2-, 3-, 4-year OS: 62%, 40%, 23%, 14%	ctDNA clearance correlated with OS; rash and normal LDH linked to longer OS
Hassel et al. (2023) [12]	Clinical trial, phase III	Tebentafusp vs. investigator’s choice—3 years observation	378	OS: 21.6 mo vs. 16.9 mo; 3-year OS: 27% vs. 18%	Common AEs: rash (83%), fever, pruritus, hypotension; 2% discontinued due to AEs
Nathan et al. (2021) [18]	Clinical trial, phase III	Tebentafusp vs. investigator’s choice—1 year observation	378	1-year OS: 73% vs. 59%; 6-mo PFS: 31% vs. 19%	HR for death: 0.51; tebentafusp superior to ICI/chemo
Rodrigues et al. (2024) [19]	Prospective cohort study	Tebentafusp	69	ORR: 10%, PFS: 2.8 mo, OS: 21.8 mo	ctDNA clearance = OS 34.6 mo vs. 12.7 mo (*p* = 0.003); predictive biomarker value
Roshardt Prieto et al. (2024) [20]	Retrospective cohort	Tebentafusp	19	ORR: 10%, PFS: 2.8 mo, OS: 18.8 mo	RECIST vs. PERCIST: discordant; PET more sensitive early
Tomsitz et al. (2024) [25]	Prospective single-center cohort	Tebentafusp	33	Skin AEs: 79%; OS 34 mo vs. 4 mo (*p* < 0.001)	CD8+ T-cell infiltration in skin; survival linked to rash
Tomsitz et al. (2023) [26]	Retrospective multi-center cohort	Tebentafusp	78	PFS: 3 mo, OS: 22 mo	ICI → tebentafusp better outcomes; sequence matters
Staeger et al. (2025) [27]	Prospective biomarker cohort	Tebentafusp	11	AEs linked to cytotoxic dermatitis,	GVHD-like features; Trm CD8+ cells; OS benefit with skin AEs
Maurer et al. (2024) [14]	Retrospective cohort	Tebentafusp vs. ICI	22 vs. 14	OS: 18.6 mo (T) vs. 28.9 mo (ICI); PFS ~2.7–2.9 mo	Response in liver mets correlates with global outcome
Dimitriou et al. (2025) [28]	Retrospective multi-center sequencing study	Tebentafusp vs. ICI sequence	131	OS: 33.6 mo (ICI → T) vs. 22.4 mo (T → I)	ICI first = better PFS/OS; prior treatment/LDH affects outcomes
Vitek et al. (2024) [29]	Retrospective multi-center cohort	Tebentafusp	23	ORR: 23%, DCR: 64%, 1-year OS: 66%	No link between response and LDH, AP, ECOG, or site
López et al. (2025) [30]	Phase I, first-in-human dose-escalation	IMCnyeso (NY-ESO-1 TCR)	7 (melanoma, including 3 MUM)	ORR: 0%, SD: 14%, PD: 71%	Better in synovial sarcoma; limited efficacy in melanoma

**Table 5:** TIL therapy in UM results.

Study	Phase	Intervention Type	Patients	Key Outcomes Reported	Selected Remarks
Monberg et al. [31]	phase I	TIL + oncolytic AdV TILT-123	17 metastatic CM/UM	ORR 11.7%	Safe across dose levels; 5 long-term survivors
				1 CR, 1 PR	
				4 SD	
				median PFS 78 d	
Lövgren et al. [32]	phase I/II	TIL + autologous dendritic-cell vaccine	14 CM/ UM	2 ongoing CR	PET/CT showed deep regressions; well tolerated
				1 durable PR	
				1 short-PR	
Nguyen et al. [33]	phase II	TIL + low-dose s.c. IL-2	12 heavily pre-treated CM/ocular melanoma	3 PR (25%)	Lower IL-2 ↓ toxicity; responses early but short
				median PFS 5.1 mo	
				median OS 6.2 mo	
Chandran et al. [34]	phase II	High-dose IL-2–supported TIL ACT	27 screened and 21 treated MUM	ORR 33%	In vitro tumor-reactive T-cell frequency predicted response
				1 ongoing CR	
				6 PR	
				2 durable SD	

**Table 6:** Oncogenic viruses in UM results.

Study	Phase	Intervention Type	Patients	Key Outcomes Reported	Selected Remarks
Smith et al. [35]	phase I	Oncolytic VSV-IFNβ-TYRP1	12 pre-treated MUM	0 CR/PR	Dose-dependent TYRP1-specific T-cell responses; epitope spreading
				4 SD	
				8 PD	
Lutzky et al. [36]	phase Ib	Coxsackievirus A21 (V937) + ipilimumab	11 MUM	best overall: 3 SD, 8 PD	Only 1 patient remained on therapy, rapid PD common
Garcia et al. [37]	Phase I	Oncolytic AdV ICOVIR-5	13 CM/UM (11 evaluable)	no objective responses	UM patients survived 3.7 × longer than matched CM
				SD in 2/5 low-dose	
				SD in 5/6 high-dose	

**Table 7:** Most common adverse events.

Treatment Option	AEs	In How Many Studies	Highest Grade Reported
T-cell engagers	rash	10	4–3
	pyrexia	10	3
	CRS	9	3
	pruritus	8	2–1
	hypotension	7	2–1
	nausea/vomiting	6	3
	fever and chills	5	1
	liver-enzyme elevations	4	4–3
	abdominal pain	2	2–1
	erythema	2	2–1
	periorbital edema	1	2–1
TILs	fever and chills	2	3
	fatigue/nausea	2	2–1
	hematologic toxicities	2	4–3
	creatine elevation	2	2–1
	liver-enzyme elevations	2	2–1
Oncogenic viruses	fever and chills	3	2–1
	diarrhea	3	2–1
	fatigue/nausea	3	2–1
	hematologic toxicities	2	4–3
	liver-enzyme elevations	2	3
	CRS	1	2–1

## Data Availability

No new data were created or analyzed in this study.

## Abbreviations

The following abbreviations are used in this manuscript:

ACT	Adoptive-cell therapy
AE	Adverse event
ALP	Alkaline phosphatase
BOR	Best overall response
BTCE	Bispecific T-Cell engager
CAR-T	Chimeric antigen receptor T Cell
CM	Cutaneous melanoma
CNS	Central nervous system
CR	Complete response
CRS	Cytokine-release syndrome
ctDNA	Circulating-tumor DNA
CTLA-4	Anti-cytotoxic T-lymphocyte-associated protein 4
DC	Dendritic cell
DCR	Disease control rate
DLT	Dose-limiting toxicity
gp100	Glycoprotein 100
GVHD	Graft-versus-host disease
HLA	Human leukocyte antigen
HR	Hazard ratios
ICI	Immune checkpoint inhibitor
IL-2	Interleukin-2
immTACs	Immune-mobilizing monoclonal Tcrs against cancer
LDH	Lactate dehydrogenase
MET	Mesenchymal–epithelial transition factor
MM	Metastatic melanoma
MTR	Metabolic tumor regression
MUM	Metastatic uveal melanoma
ORR	Objective response rate
OS	Overall survival
OV	Oncolytic virus
PD-1	Anti-programmed cell death protein 1
PDL-1	Anti-programmed death ligand 1
PD	Disease progression
PERCIST	PET Response Evaluation Criteria In Solid Tumors
PFS	Progression-free survival
PR	Partial response
RECIST	Response Criteria In Solid Tumors
RP2S	Recommended phase II dose
scRNA-seq	Single-cell RNA sequencing
SD	Stable disease
SIRT	Selective internal radiotherapy
TACE	Transarterial chemoembolization
TCR	T-cell receptor
TCR-T	T-cell-receptor-engineered T-Cell
TIL	Tumor-infiltrating lymphocyte
TMB	Tumor mutational burden
TME	Tumor microenvironment
TRAEs	Treatment-related adverse events
Trm	CD103^+^ Tissue-resident memory
TYRP-1	Tyrosinase-related protein 1
UM	Uveal melanoma
UMIS	Uveal Melanoma Immunogenomic Score
VSV	Vesicular stomatitis virus
